# 18-Fluorine-Fluorodeoxyglucose Positron Emission Computer Tomography Imaging in Melioidosis: Valuable but Not Essential

**DOI:** 10.3390/tropicalmed10030069

**Published:** 2025-03-06

**Authors:** Joshua Bramwell, Natalia Kovaleva, Joshua J. Morigi, Bart J. Currie

**Affiliations:** 1 Infectious Diseases Department, Royal Darwin Hospital, Darwin, NT 0810, Australia; joshua.bramwell@monashhealth.org; 2 Infectious Diseases Department, Monash Health, Melbourne, VIC 3168, Australia; 3 Division of Nuclear Medicine, Royal Darwin Hospital, Darwin, NT 0810, Australiajoshua.morigi@nt.gov.au (J.J.M.); 4 College of Medicine and Public Health, Flinders University, Adelaide, SA 5042, Australia; 5 Global and Tropical Health Division, Menzies School of Health Research, Charles Darwin University, Darwin, NT 0811, Australia

**Keywords:** melioidosis, *Burkholderia pseudomallei*, 18-F FDG PET/CT, pyrexia of unknown origin

## Abstract

Melioidosis is an endemic tropical disease caused by *Burkholderia pseudomallei*. It typically causes pulmonary disease and bacteraemia but can disseminate to cause multi-organ disease. 18-F FDG PET/CT has an evolving role in diagnosing other infectious diseases, especially where the pathogen or extent of infection is challenging to elucidate clinically and with conventional imaging (CT, US and MRI). We present a case series of patients diagnosed with melioidosis who also underwent 18-F FDG PET/CT from December 18th 2018 to September 30th 2022. Indications for imaging were categorised and analysed as to whether 18-F FDG PET/CT changed management over conventional imaging. Twenty-one 18-F FDG PET/CT scans were performed for sixteen patients. Two scans (9.5%) performed for pyrexia of unknown origin changed management in both cases. Twelve scans (57.1%) performed to ascertain the extent of dissemination of melioidosis changed management in only three (25%) cases. Five scans (23.8%) performed to monitor the response to treatment of known foci changed management in all five cases. Five scans (23.8%) performed for suspected or known malignancy changed management in three (60%) cases. 18-F FDG PET/CT is an emerging tool which improves diagnosis and changes the management of melioidosis when applied judiciously and for well-selected indications.

## 1. Introduction

Melioidosis is an infectious disease caused by *Burkholderia pseudomallei*, a Gram-negative bacterium which can infect humans and a wide range of animals. *B. pseudomallei* is commonly found in soil and surface groundwater and is endemic to tropical areas, especially in northern Australia and Southeast Asia [1]. Infection is acquired via exposure through damaged skin, inhalation or ingestion and usually results in subclinical disease that is cleared by immunocompetent hosts. *B. pseudomallei* infection that causes symptoms is termed melioidosis, with the majority of patients (88%) presenting with an acute infection, and less commonly chronic infection (9%) and reactivation of latent infection (3%) [2,3]. Bacteraemia occurs in over half of all patients with melioidosis, and the most common clinical presentations are pneumonia, skin infection and genitourinary infection, with less common presentations including soft tissue abscesses, septic arthritis, osteomyelitis and neurological infection [4].

18-Fluorine-Fluorodeoxyglucose Positron Emission Computer Tomography (18-F FDG PET/CT) has an increasing role in the investigation and monitoring of infectious diseases, with applications in pyrexia and bacteraemia of unknown origin, *Staphylococcus aureus* bacteraemia, as well as prosthetic valve infective endocarditis and vascular graft infections [5,6,7,8,9,10]. In this context, 18-F FDG PET/CT has had utility in identifying and monitoring foci of disseminated infection, suggesting that it may have a role in melioidosis as well, where the location and extent of foci are often not readily apparent and where the monitoring of these foci over a prolonged treatment course is essential.

The role of 18-F FDG PET/CT in the investigation of melioidosis is not defined in the literature but has been described in case reports and small case series involving patients presenting with pyrexia of unknown origin to identify foci of disseminated melioidosis [11]. Other case series have found utility in identifying both symptomatic and asymptomatic sites of infection in patients with known melioidosis [12,13]. It has also been used serially to assess treatment efficacy where other imaging modalities are confounded by residual post-infective changes [11]. 18-F FDG PET/CT has also been used in the workup of lesions suspicious for malignancy which, on biopsy, ultimately cultured *B. pseudomallei* [14,15].

The Darwin Prospective Melioidosis Study began in October 1989, and 18-F FDG PET/CT first became available at Royal Darwin Hospital on 5 December 2018. We investigated all 18-F FDG PET/CT scans performed from this date to the last case of melioidosis in the 2021-22 wet season, which occurred on 30 September 2022, with the aim to determine the diagnostic and management impacts of 18-F FDG PET/CT in patients with melioidosis.

## 2. Materials and Methods

We included all patients with melioidosis confirmed on blood, sputum, urine, fluid or tissue culture who also had 18-F FDG PET/CT performed between 5 December 2018 and 30 September 2022. The indications for 18-F FDG PET/CT were determined, and timing and comparisons with other radiology and patient clinical course were analysed. Therapy for cases and duration of intravenous and oral eradication phases were as in the 2020 Darwin melioidosis treatment protocol [16].

This study was approved by the ethics committee of the Northern Territory Department of Health and Menzies School of Health Research (approval number 02/38). A waiver of consent from individual participants was granted. Data were accessed for research purposes only and included only deidentified patient information. All methods were performed in accordance with the relevant guidelines and regulations. This study did not receive funding.

## 3. Results

There were 192 melioidosis cases between 5 December 2018 and 30 September 2022, 10 (5.2%) of which died due to melioidosis and 25 (13.0%) of which had one or more 18-F FDG PET/CT procedures performed for any reason. Upon review, nine of the 18-F FDG PET/CT scans performed were entirely unrelated to the diagnosis or management of melioidosis and were excluded from our study. Notably, we included patients who had 18-F FDG PET/CT performed for the diagnosis or management of malignancy but where the PET diagnosed or changed the management of their melioidosis. We therefore analysed the 21 18-F FDG PET/CT scans performed for the remaining 16 patients, with some 18-F FDG PET/CT scans having multiple indications.

The indications for 18-F FDG PET/CT were placed into four categories:

1. Two 18-F FDG PET/CT scans performed for pyrexia of unknown origin (9.5%);
2. Twelve 18-F FDG PET/CT scans for ascertaining the extent of dissemination of melioidosis (57.1%);
3. Five 18-F FDG PET/CT scans for monitoring the response to treatment of known foci (23.8%);
4. Five 18-F FDG PET/CT scans for suspected or known malignancy (23.8%).

Demographics, comorbidities, clinical scenarios, indications for 18-F FDG PET/CT, the results of 18-F FDG PET/CT and the changes in management (if any) for the 16 cases are summarised in Table A1.

For each 18-F FDG PET/CT indication, it was determined whether the 18-F FDG PET/CT changed management based on the following:

1. Pyrexia of unknown origin 2/2 (100%);
2. The extent of dissemination of melioidosis 3/12 (25%);
3. The monitoring response to the treatment of known foci 5/5 (100%);
4. Suspected or known malignancy 3/5 (60%).

**Figure 1:**
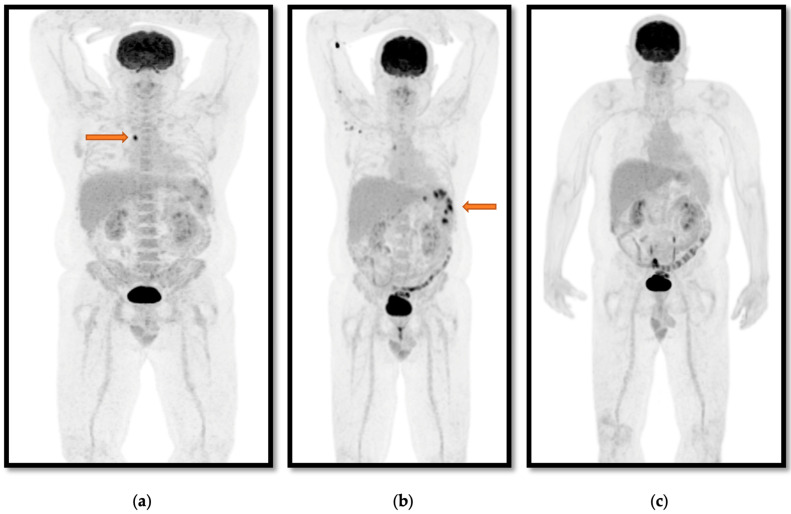
An illustrative case (case 8) of a patient who presented with pyrexia of unknown origin despite undergoing blood cultures and CT. (**a**) 18-F FDG PET/CT identifying an FDG avid lymph node (arrow), which was biopsied and cultured *B. pseudomallei*. (**b**) The result of 18-F FDG PET/CT performed after completing 4 weeks of intensive phase therapy as the patient remained febrile. This showed increased FDG avidity of splenic foci (arrow), extending the IV intensive therapy phase to 12 weeks. (**c**) The result of 18-F FDG PET/CT performed due to persistent symptoms at 30 weeks of treatment, showing complete resolution of foci, giving clinicians and the patient confidence to cease antibiotics.

When 18-F FDG PET/CT was performed for pyrexia of unknown origin, it changed management by prompting biopsy in both cases. In case 6 (Table A1), it prompted biopsy of an 18-F FDG PET/CT avid lesion, which cultured *B. pseudomallei* 26 days after presenting to the hospital and subsequent to three CT scans and one MRI over the 26 days. In case 8 (Table A1, Figure 1), CT identified multiple splenic lesions; however, 18-F FDG PET/CT identified an 18-F FDG PET/CT avid lymph node not identified on CT, prompting a lymph node biopsy, which was culture-positive for *B. pseudomallei,* enabling the diagnosis of melioidosis.

18-F FDG PET/CT performed for determining the extent of dissemination was helpful in a minority of cases, changing management in only 3 of 12 18-F FDG PET/CT scans. Case 15 (Table A1) provides an example of circumstances in which 18-F FDG PET/CT identified metabolic activity in prosthetic material at the thoracic endovascular aortic repair (TEVAR) site not identified on CT angiography after a positive blood culture for *B. pseudomallei*, clarifying the need for lifelong suppressive antibiotics. It also identified a splenic infarct suggesting septic emboli and suggesting to clinicians that the original penetrating ulcer requiring TEVAR was very likely a mycotic pseudoaneurysm. In case 10, a man with prostate and cutaneous melioidosis with poor treatment response prompted clinicians to look for further disseminated foci of melioidosis. 18-F FDG PET/CT identified possible ischial osteomyelitis underlying known cutaneous melioidosis. This extended the treatment duration to a minimum of 6 weeks [16]; however, this duration was subsequently over-ridden by ongoing urine culture positivity at 6 weeks due to an insufficiently drained prostatic abscess also seen on 18-F FDG PET/CT.

When 18-F FDG PET/CT was used to monitor treatment response, it was most helpful when treatment had already extended beyond the minimum recommended by the Darwin treatment protocol due to poor response to therapy or when the patient had persistent symptoms at the time of planned completion of intravenous treatment [16]. This was exemplified in case 2, when a 10-week induction phase of intravenous therapy (rather than 6 weeks) was chosen for osteomyelitis disseminated throughout the skeleton due to clinical and radiological progression on MRI. In addition, the oral eradication phase was also extended from 6 to 9 months due to persistent symptoms. The 18-F FDG PET/CT results showing complete resolution allowed both the clinician and the patient to feel comfortable with stopping treatment despite persistent arthralgia. Similarly in case 8, the 18-F FDG PET/CT showed increasing avidity of splenic foci at 3.5 weeks of a planned 4-week treatment period despite no volumetric change seen on CT, prompting extension of induction intravenous therapy (Figure 1). 18-F FDG PET/CT also gave clinicians confidence to transition from intensive intravenous phase to oral eradication phase in case 11, where the patient recently had a relapse of melioidosis despite having undergone treatment. Furthermore, in case 15, complete resolution of 18-F FDG PET/CT avidity at the TEVAR graft site while taking suppressive cotrimoxazole once daily suggested that this was sufficient for the long-term management of the infected graft. Notably, every patient with melioidosis who had 18-F FDG PET/CT performed to monitor treatment response already had a preceding 18-F FDG PET/CT result for comparison.

In the five cases in which 18-F FDG PET/CT was performed for the workup of suspected or known malignancy, 18-F FDG PET/CT excluded malignancy in two cases (cases 8 and 9) by prompting biopsy, which subsequently cultured *B. pseudomallei* (Figure 1). In case 1, 18-F FDG PET/CT staged the patient’s breast cancer which was previously diagnosed when the patient presented with *B. pseudomallei* breast abscess and therefore guided the oncological treatment approach. In case 4, 18-F FDG PET/CT appearance was not consistent with malignancy, and it was planned for the patient to undergo serial imaging, presenting in the interim with *B. pseudomallei* sepsis, with the 18-F FDG PET/CT not significantly changing management.

## 4. Discussion

CT and ultrasound imaging have been available for all three decades of the Darwin Prospective Melioidosis Study. MRI has only been available since 1993, and 18-F FDG PET/CT has only been available since 2018. Mortality from melioidosis in the Darwin study improved from 31% in the 1989–1994 period to 6% in the 2014–2019 period as a result of advances in the overall diagnostic and management approach of which developments in diagnostic imaging are a part [2]. In most locations where melioidosis is endemic, 18-F FDG PET/CT, MRI and, in many cases, even CT are unavailable. Our study indicates that there is likely a small but important role for 18-F FDG PET/CT when performed for specific indications to optimise the diagnosis and management of melioidosis. As suggested in the literature, 18-F FDG PET/CT has a role in pyrexia of unknown origin, and this holds true for melioidosis, guiding biopsy to isolate *B. pseudomallei* despite unhelpful CTs being performed prior. 18-F FDG PET/CT also changes management when it is performed to monitor sites of known infection, guiding clinicians when melioidosis does not clinically, biochemically or radiologically respond to treatment as expected. 18-F FDG PET/CT can help guide antibiotic duration for clinicians and can reassure patients with ongoing symptoms when treatment has extended beyond the guidelines outlined in the 2020 Darwin melioidosis treatment protocol [16]. 18-F FDG PET/CT was never performed at a time point earlier than the planned completion of the intensive or eradication phase with the intention of cutting short the duration of therapy, so from our data, we cannot draw any conclusions about using 18-F FDG PET/CT to shorten therapy. 18-F FDG PET/CT, however, adds little to conventional CT imaging in the majority of cases when used to determine the extent of infection. Our data suggest that 18-F FDG PET/CT could be reserved for specific indications such as suspected infected prostheses, where the sensitivity of CT may be limited, or for patients in which an occult uncontrolled source is clinically suspected but has not been identified using conventional imaging. It is important to note, however, that when 18-F FDG PET/CT was used to monitor infection, it was the most helpful if it was able to be compared to a prior 18-F FDG PET/CT scan performed for another indication, usually one performed to determine the extent of infection. 18-F FDG PET/CT performed for the workup of suspected or known malignancy has variable utility and is challenging to draw conclusions from; diagnosing melioidosis on biopsy was an unexpected finding. However, it highlights how melioidosis can frequently mimic malignancy radiographically and that biopsies in melioidosis endemic regions should be sent fresh for bacterial culture in addition to histology.

Our study has a number of limitations: First is its generalisability, as there are few other healthcare settings with such high incidence of melioidosis with the same availability and access to 18-F FDG PET/CT. Melioidosis is known to be endemic in northern Australia and Southeast Asia and has increasingly been identified across South Asia, Africa, the Americas and the Pacific, most frequently in low- and middle-income countries [17]. Of 192 patients diagnosed with melioidosis while 18-F FDG PET/CT was available in the Darwin study, only 16 (8.3%) underwent 18-F FDG PET/CT, and of these, only 9 (4.7% of total cases) underwent 18-F FDG PET/CT that changed management. This suggests that for the majority of cases, conventional imaging is sufficient in terms of diagnostics. The significant mortality reduction seen over the 30 years of the Darwin Prospective Melioidosis Study occurred prior to the availability of 18-F FDG PET/CT^2^.

Another limitation is the retrospective nature of this study, which requires judgements to be made regarding the expected clinical course if 18-F FDG PET/CT had not been performed. This is especially difficult to determine for 18-F FDG PET/CT scans performed at the end of a planned course of treatment for patients with ongoing symptoms. While we determined that these 18-F FDG PET/CT scans changed management in each case, this may overestimate 18-F FDG PET/CT’s utility here; a viable alternative of ceasing therapy and monitoring progress may have achieved similar results. However, this could have come at the cost of clinician and patient confidence that melioidosis had been adequately treated, with the results of 18-F FDG PET/CT providing reassurance, especially in patients who had previously had relapse of melioidosis or had experienced prolongation of treatment to over 1 year.

## 5. Conclusions

In conclusion, this study demonstrates the key indications for which 18-F FDG PET/CT changes the diagnosis and management of melioidosis compared to conventional imaging. This method most notably facilitates pathogen identification in pyrexia of unknown origin via biopsy and guides management when treatment has already extended beyond treatment guidelines due to poor response to planned therapy. It also supports the strength of conventional imaging, primarily with CT, in identifying the extent of infection to determine treatment duration, with 18-F FDG PET/CT being especially useful if there is strong clinical suspicion of an uncontrolled source despite an unrevealing CT.

## Data Availability

All data relevant to the article are included in the article. Further inquiries can be directed to the corresponding authors upon reasonable request.

## Abbreviations

The following abbreviations are used in this manuscript:

PET	Positron Emission Tomography
FDG	Fluorodeoxyglucose
CT	Computed Tomography
US	Ultrasound
MRI	Magnetic Resonance Imaging
TEVAR	Thoracic Endovascular Aortic Repair

## Appendix A

**Table A1:** Demographics, comorbidities, clinical scenarios, indications for 18-F FDG PET/CT, results of 18-F FDG PET/CT and changes in management (if any). If 18-F FDG PET/CT, results changed management this has been highlighted in green, and if it did not this is highlighted in red.

Demographics and Comorbidities	Clinical Scenario	Indication Category	18-F FDG PET/CT Result	Change in Management
1. 69F T2DM, CKD, IHD, asthma	Previous pulmonary melioidosis, represented with concurrent breast cancer and *B. pseudomallei* breast abscess	1st PET: Extent of dissemination AND suspected/known malignancy	1st PET: Study identified breast lesion with no disseminated or metastatic disease	1st PET: Did not change management of melioidosis 1st PET: Guided oncological management approach
2. 56F T2DM	Disseminated melioidosis with pulmonary, splenic, liver, peripancreatic and widespread upper and lower limb osteomyelitis with multiple septic joints	1st PET: Extent of dissemination 2nd PET: Monitoring response to treatment	1st PET: Identified extent of infection but did not add to previous MRI results 2nd PET: Performed 9 months later; showed resolution of PET avidity	1st PET: Did not change management over clinical examination and MRI findings 2nd PET: Gave treating clinicians confidence to cease treatment despite ongoing pain thought to be due to mechanical arthritis
3. 81M Myelodysplastic syndrome, CKD, IHD	Initially thought to be *B. pseudomallei* bacteraemia with no focus as it had unremarkable CT CAP results	1st PET: Extent of dissemination	1st PET: Multiple FDG avid nodules in lung, mediastinum and duodenum	1st PET: Identified pulmonary involvement not seen on CT 2 weeks earlier, altering duration of IV intensive phase of therapy
4. 73F T2DM, asthma/COPD, cognitive impairment	Presented 1 month prior to diagnosis of melioidosis with pneumonia and lung lesion, improved and discharged home, PET ordered for malignancy investigation given persistent lung lesion; represented following PET with respiratory sepsis	1st PET: Suspected/known malignancy	1st PET: Partially cystic lung lesion thought unlikely to be malignant; no metastatic or disseminated disease	1st PET: Did not change management compared to conventional imaging
5. 42M Asthma Fibula ORIF	Disseminated melioidosis with pulmonary splenic and prostatic and cutaneous abscesses Clinical concern for infected metalware at ORIF site	1st PET: Extent of dissemination	1st PET: PET performed 2 months after diagnosis; showed active prostate infection, no other foci of infection	1st PET: Did not change management compared to conventional imaging and clinical impression.
6. 31F T2DM, RHD	Disseminated melioidosis with preceding pyrexia of unknown origin; presented with back pain and rising melioidosis serology titre; subcarinal lymphadenopathy identified on CT	1st PET: Pyrexia of unknown origin	1st PET: Identified multiple nodal, splenic and small bowel foci	1st PET: Prompted lymph node biopsy; identified small bowel and splenic FDG avid lesions that were not identified on CT or abdominal ultrasound; guided duration of IV intensive therapy
7. 29F Hazardous alcohol use	Disseminated melioidosis with bacteraemia, scalp and extradural abscess	1st PET: Extent of dissemination	1st PET: Large left temporal melioidosis site and few subcutaneous and cervical nodularities; no distant foci	1st PET: Did not change management of conventional imaging with CT and MRI
8. 50M T2DM, hazardous alcohol use	Disseminated melioidosis with splenic hepatic and nodal foci; presented with pyrexia of unknown origin, arthralgia and abdominal pain; CT findings of disseminated lesions concerning for metastatic malignancy	1st PET: Pyrexia of unknown origin AND suspected/confirmed malignancy 2nd PET: Monitoring response to treatment 3rd PET: Monitoring response to treatment	1st PET: FDG avid paratracheal lymphadenopathy and splenic foci 2nd PET: New axillary FDG avid axillary lymph nodes and increased avidity of known splenic foci 3rd PET: No positive foci of infection identified	1st PET: Identified new foci which guided biopsy, leading to diagnosis of melioidosis 2nd PET: Prompted extension of IV intensive phase from 4 weeks to total of 12 weeks due to increasing SUVmax of splenic foci at 3.5 weeks of IV therapy; CT performed prior to PET showed that these lesions were volumetrically unchanged 3rd PET: Gave clinicians confidence to cease antibiotics at planned duration despite ongoing non-specific symptoms
9. 68M T2DM	Concurrent melioidosis and tuberculosis diagnosed after positive PET and lymph node biopsy performed during investigation for hoarse voice initially presumed to be due to cancer	1st PET: Suspected/confirmed malignancy	1st PET: Low-grade mediastinal and cervical lymphadenopathy	1st PET: Identified foci were limited to lymphadenitis in mediastinum and cervical lymph nodes guiding duration of therapy
10. 49M CKD3b, urethral stricture, COPD, latent TB, chronic hepatitis B, right AKA due to previous necrotising fasciitis, anal SCC in remission	Disseminated melioidosis with osteomyelitis, prostatic and cutaneous foci; relapsed 18 months later with prostatic, splenic and hepatic abscesses	1st PET: Extent of dissemination	1st PET: Identified prostatic, ischial, stomach and colonic foci	1st PET: Identified bony FDG avidity suggestive of osteomyelitis not previously identified on other imaging, which guided planned duration, but duration extended beyond this due to persistently positive urine cultures
11. 65F T1DM, HFrEF, pulmonary HTN	Pulmonary melioidosis with incomplete eradication phase; relapse representation 4 months later with 1st PET performed at that time and 2nd performed 6 weeks later	1st PET: Extent of dissemination 2nd PET: Monitoring response to treatment	1st PET: Bilateral pulmonary and mediastinal nodal foci; identified nonspecific uptake in small bowel 2nd PET: Near resolution of pulmonary and mediastinal foci; resolution of small bowel focus	1st PET: Did not change management compared to chest CT 2nd PET: Gave clinicians confidence to transition to oral eradication phase therapy given previous relapse
12. 45F T2DM, asthma/COPD	Pulmonary melioidosis with pleural effusion; delay in isolating *B. pseudomallei* with multiple courses of ineffective antibiotics	1st PET: Extent of dissemination	1st PET: No hypermetabolic findings to suggest active melioidosis	1st PET: Did not change management over CT
13. 66M T2DM, CKD3a, multiple myeloma	Pulmonary melioidosis concurrent with active treatment with lenalidomide for multiple myeloma	1st PET: Extent of dissemination AND suspected/known malignancy	1st PET: Multiple hypermetabolic lesions in mediastinal lymph nodes and skeleton	1st PET: Did not change management compared to CT
14. 71M Hazardous alcohol use, CP-A cirrhosis	Cutaneous melioidosis with dissemination and subsequent osteomyelitis	1st PET: Extent of dissemination	1st PET: Active soft tissue and possible osteomyelitis	1st PET: Did not change management compared to CT; MRI was only modality that clearly showed evidence of osteomyelitis
15. 65M No comorbidities	Underwent TEVAR for penetrating thoracic aortic ulcer 10 days prior to diagnosis of bacteraemic melioidosis after representing febrile	1st PET: Extent of dissemination 2nd PET: Monitoring response to treatment	1st PET: Active infection with paraortic and retrocrural collections 2nd PET: Complete metabolic response to antibiotic therapy	1st PET: Confirmed clinical suspicion of graft infection not identified on CT 2nd PET: Complete resolution of metabolic changes while on lifelong suppressive cotrimoxazole, suggesting adequate suppression
16. 71F Bronchiectasis	Pulmonary melioidosis after presenting with 6-month history of productive cough	1st PET: Extent of dissemination	1st PET: No hypermetabolic active foci of infection	1st PET: Did not change management of melioidosis compared to CT
